# Welsh Sanatorium Construction and Equipment

**Published:** 1914-02-14

**Authors:** 


### WELSH SANATORIUM CONSTRUCTION AND EQUIPMENT.

### A Discussion at the Sanitary Institute.

At the usual sessional meeting of the Royal Sanitary Institute on Tuesday, February 10, Mr. David Davies, M.P., in the chair, Mr. Edwin T. Hall, F.R.I.B.A., described in detail, aided by some seventy sheets of drawings, the new King Edward VII. Sanatorium, which is to be erected at Pont-y-Wall, near Talgarth, South Wales.

The Chairman briefly sketched the history of the movement, which originated in the desire of the National Memorial Association in Wales to erect a suitable memorial to the late King Edward. It was felt that there could be no more fitting memorial than a crusade against tuberculosis, a disease which carries off more people in Wales than in any other part of the United Kingdom. When the Association came into existence three years ago it proceeded to devise a very comprehensive scheme, which should comprise the education of the people concerning tuberculosis and kindred diseases, and the erection of sanatoria and hospitals. For three years lectures had been given to school children and the public generally, and a travelling exhibit had been touring the country with the same object, *i.e.* instilling the basic laws of health and hygiene. Schemes and plans had already been secured for at least twelve hospitals, which it was hoped would be erected during the next year; and meantime accommodation had been secured at some fever and smallpox hospitals. The third line of attack was by erecting two large sanatoria for the Principality; one in the North, and the other in the South. The first would contain 150 beds, and that in the South 250 beds for adults and fifty for children. At the outset it was difficult to find the necessary financial resources, but the Insurance Act provided sufficient funds, and the local authorities and County Councils had, with one exception, joined the Association in carrying out the scheme, which had been co-ordinated under a Board of Governors and a Council representing the County Councils and Insurance Committees all over Wales. Considerations of efficiency and economy led to the determination to have two large sanatoria, rather than a number of small ones at different places. The services had been secured of Dr. Marcus Paterson, who had done distinguished work at the Frimley Sanatorium. He recently heard a distinguished politician say that sanatoria, after they had served their purpose for ten years, should be burned down. That reflected the view held by many people to-day: that sanatoria and tuberculosis hospitals were places full of infection, and it was difficult to combat that idea in the minds of the public, and show that those places were much less infectious than the ordinary railway carriage or public meeting-room. The buildings which Mr. Hall was about to describe would be permanent, and when tuberculosis had been eradicated they could be used for other diseases, or for the treatment of the convalescent sick. He was a member of the Departmental Committee, which stated in its report that sanatoria should be erected at a cost of £150 per bed. He disagreed from that view, and no doubt Mr. Hall would show that it was almost impossible, having regard to due efficiency, to erect a satisfactory institution at that cost.

Mr. Hall said this institution about to be built was the largest and one of the first—if not the first—sanatorium to be erected under the National Insurance Act. The site was an ideal one of 375 acres, six miles from Brecon, sloping generally to the south and commanding beautiful views of the Black Mountains. The 304 beds would be allocated as follows:

| | |
|---|---|
| Male ambulant patients ... ... ... | 125 |
| Female ditto ... ... ... ... ... ... | 75 |
| Children ... ... ... ... ... ... ... | 48 |
| Children in isolation ... ... ... ... | 6 |
| Males and females requiring special nursing | 50 |

The staff would number 73, so that the number to be housed totalled 400. The sexes would be rigidly separated from each other. With strict economy always in mind the buildings, while of fire-resisting construction throughout, were of the simplest elements. The pavilions were of ferro-concrete with three-inch external walls; plastering and glazed brickwork were omitted generally. The nurses' home was placed south of the sanatorium, so that when off duty they would be well away from "shop." The drainage would be by a duplicated system—one main pipe for soil and another for rain-water. The soil would be carried to an outfall tank and treated, the effluent being taken to the stream on the estate. Rain-water would be stored for use in the boilers. The drinking water would be supplied from Talgarth, and be drawn direct from the main. The lighting and the power for driving all machinery would be by electricity, generated in the power-house. Steam boilers would be in duplicate, each capable of doing the whole required work, and an accumulator plant was in contemplation; it would enable the institution to be run at night without keeping the engines going. It was proposed to heat, by means of steam pipes and radiators, the nursing block, the children's pavilions, the patients' dining and waiting halls, the staff dining rooms, and the corridors of men's quarters. There would be no heat in the ambulant wards or workshops; many of the patients would be out of doors and at work; and in former institutions where heating means were provided they were scarcely ever used; patients did not want them. There was a 700 feet covered way. A sterilising plant would be provided for clothes and sputum; and there would be a laboratory, *post-mortem* room, mortuary, boot-repairing shop, workshops, etc., and large stores to provide against impassable roads, the occurrence of strikes, etc.

The great problem had been to erect an efficient and a sufficient building at a very low price. The Departmental Committee, in clause 4 of the introduction to their Report, recommended "grants up to three-fifths of the cost per bed (which is estimated at not exceeding £150) should be made for the provision of additional sanatorium and hospital beds for adults, provided that the total sum should not exceed £90 per bed." This, said Mr. Hall, had been interpreted to include land and furniture. Sanatoria had been erected in this country at a cost of £300 to even £1,000 per bed. The task laid down in this Report was so inelastic as to be impracticable; and the inclusion of land in the figure meant getting as little land as possible, whereas plenty of open space was an important desideratum. He did not say that sanatoria of thirty to sixty beds could not be built for the money if there were a limited area of land, and if plain concrete sheds, with the minimum administrative buildings, earth closets, and no machinery, boilers, electric light, etc., were installed; but no large sanatorium designed to comply with the requirements of the Departmental Sub-Committee could be erected at a cost of £150 per bed. Benenden Sanatorium, at a cost of £100 per bed, was often quoted; but that proved to be an illusion, for of its 105 beds, twenty were a free gift, and the total cost of the other eighty-five up to last year was marked out at £213 per bed, without including land or furniture, and the sanatorium had not electric lighting or generating plant, laundry, or covered way between pavilions. It had been found that engineering plant of all sorts cost about 33 per cent. of the whole, and the cost of the patients' pavilions was about the same. In accordance with what was laid down, the rooms would be made only 8 ft. square. For the ambulance cases that would mean that it was only a sleeping apartment. Attached to each dining-room was a washing-up room, because under Dr. Paterson's scheme each individual was responsible for his or her washing-up. There would likewise be reading- and writing-rooms for each sex, and provision for entertainments. The cost of the buildings, it was hoped, would work out at something like £150 per bed, excluding land and furniture. If £150 per bed should have been realised he thought it would constitute a record for a big institution complete in every way.

Dr. Robertson (Medical Officer of Health, Birmingham), in discussing the subject, said he regarded this as the best example of English sanatorium construction; our models were becoming very different from those in other countries, notably America, Germany, and Switzerland. Eight years ago Birmingham erected a sanatorium sixty miles away in the Cotswolds, but later set up one near the town, and the latter had been far the more successful and had a greater educative value than the other, as the visiting relatives, who attended freely, could easily be taught important facts by means of lectures. He agreed that though half an acre per bed was good for small institutions, yet for large sanatoria anything more than about one quarter an acre per bed was not only undesirable but a nuisance. He agreed there should be no heating of the pavilions; in one institution where stoves were placed they were practically never used. Phthisis patients not only could stand a great deal of air in strong currents, but enjoyed it. Gas, where there were so many draughts, was a failure; electricity was the only practical illuminant. If the scheme had allowed it, he would have liked to see terrazzo floors. In his experience, compulsory notification had brought as many women as men patients, and in the case of the women they had not the fear of losing their employment to the same extent as the breadwinner of the family had.

Mr. Alfreton Smith thought measures now planned for fighting it were to some extent a "peddling" with the question. He congratulated Mr. Hall on what he was producing for the money, and compared it with the comparative luxury and sense of prosperity pervading Frimley Sanatorium.

Dr. Louis Parkes (Medical Officer of Health, Chelsea) said the sanatorium treatment for London was not being carried out thoroughly: it was done in places which had been built for something else. In his district patients stayed on an average of two and a half months. He agreed that sanatoria for towns should be close to the towns. It was easy to ride economy of cost too hard in this matter. Some of the earlier sanatoria were admittedly far too expensive, but working people should not be required to go to places which were little better than cattle-sheds. He thought there should be some means of heating the sleeping apartments when necessary; the weather was sometimes so severe that it would, especially on such an exposed site, freeze the water in hot-water bottles during the night. Those who, in the Departmental Committee, advocated such cheap structures, should try residence in them. Money now being spent on sanatoria would have been much better used years ago in schemes of proper housing and sanitation, and in paying adequate wages to the masses of the people.

